# Noble gases xenon and argon: from cellular signalling mechanisms to organoprotection and clinical applications

**DOI:** 10.1186/s12967-026-07944-2

**Published:** 2026-03-09

**Authors:** Qian Chen, Shifan Zhu, Minghui Wu, Jiashi Sun, Moradi Kimia, Dinayinie Ekanayake Mudiyanselage, Hakjun Lee, Daqing Ma

**Affiliations:** 1 Perioperative and Systems Medicine Laboratory and Department of Anesthesiology, Children’s Hospital, Institute of Translational Medicine, Zhejiang University School of Medicine, National Clinical Research Center for Children and Adolescent’s Health and Diseases, Zhejiang, China; 2 Department of Anesthesiology, The First Affiliated Hospital, Ningbo University, Ningbo, China; 3 Division of Anaesthetics, Pain Medicine and Intensive Care, Department of Surgery and Cancer, Faculty of Medicine, Imperial College London, Chelsea & Westminster Hospital, London, UK

**Keywords:** Xenon, Argon, Noble gas, Anesthesia, Organ protection

## Abstract

**Background:**

Noble gases xenon (Xe) and argon (Ar) emerge as promising therapeutic agents. Extensive studies have validated their efficacy across various models of organ injury, positioning them as novel candidates for clinical translation in critical care and perioperative medicine.

**Main Body:**

Xe and Ar exert protective effects through multiple mechanisms, including activation of hypoxia-inducible factor-1 (HIF-1) pathway, inhibition of regulated cell death pathways, such as apoptosis, necroptosis, ferroptosis, and pyroptosis, and suppression of pro-inflammatory signaling. By modulating these key signaling pathways, Xe and Ar have been shown to improve outcomes in neurological, cardiac, renal, and hepatic systems across diverse models of ischemia-reperfusion injury, traumatic brain injury, and systemic inflammation. Clinically, Xe has shown efficacy in anesthesia, neonatal neuroprotection, and cardiac arrest management. Ar, with greater availability and lower costs, holds promise for broader clinical use but remains in the early stage of translational research.

**Conclusion:**

Xe and Ar represent novel biologically active gases with the potential to provide promising therapies in perioperative and clinical care medicine. Overcoming current limitations, such as a lack of standardized delivery systems and optimized dosing strategies, is key to uncovering their clinical application.

## Introduction

Noble gases, including helium, neon, argon (Ar), krypton, xenon (Xe), and radon, are characterized by complete valence electron shells that confer exceptional stability and lack of reactivity under physiological conditions. While historically considered biologically inert, recent research has revealed their diverse pharmacological effects, particularly in anesthesia, neuroprotection, and graft preservation [1–3]. Specifically, Xe and Ar have garnered significant attention due to their unique physicochemical properties, which further confer notable therapeutic potential, making them a focus of interest in relevant research and clinical exploration.

Xe, with an atomic number of 54 and atomic mass of 131.29 u, is one of the heaviest noble gases and is present in the atmosphere at a concentration exceeding 0.0875 ppm. Its solubility in water is moderate (~108 mL/L) [4], making it suitable for biomedical research uses. Xe is non-toxic, eliminated entirely *via* exhalation, and has been shown to have anesthetic and neuroprotective effects in both animal and human studies [5–8]. Ar, with an atomic number of 18 and an atomic mass of 39.95 u, possesses two key properties: it is lighter and more abundant in the atmosphere. These two features work in tandem. Argon’s high atmospheric abundance ensures easy and economical sourcing, and its low density adapts to diverse industrial operational needs, making it widely used in industrial applications [9]. In contrast with Xe, Ar requires specific conditions (e.g., with highly electronegative elements) to form stable compounds [10]. Its ability to modulate cell signaling without significant metabolism in the body makes it a potential therapeutic gas [11, 12].

## Pharmacodynamics of Xe and Ar

### Activation of hypoxia-inducible factor-1 (HIF-1) system

The HIF-1 system is essential for cellular adaptation to hypoxic and ischemic environments, primarily regulating genes involved in oxygen delivery, metabolism, and survival pathways. HIF-1 is a heterodimeric transcription factor composed of HIF-1α, an oxygen-sensitive subunit, and HIF-1β [13]. Under normoxic conditions, HIF-1α undergoes hydroxylation by prolyl hydroxylase domains (PHDs), marking it for proteasomal degradation. The enzymatic activity of PHD is critically dependent on molecular oxygen as a substrate. Hypoxia, by depriving this essential co-factor, stablizes Hif-1α and facilitates its nuclear translocation. In the nucleus, HIF-1α dimerizes with HIF-1β to bind hypoxia-responsive elements (HREs) in target genes, such as vascular endothelial growth factor (VEGF) and erythropoietin, thereby promoting transcriptional programs that enhance oxygen delivery, metabolism, and angiogenesis [14, 15].

**Fig. 1:**
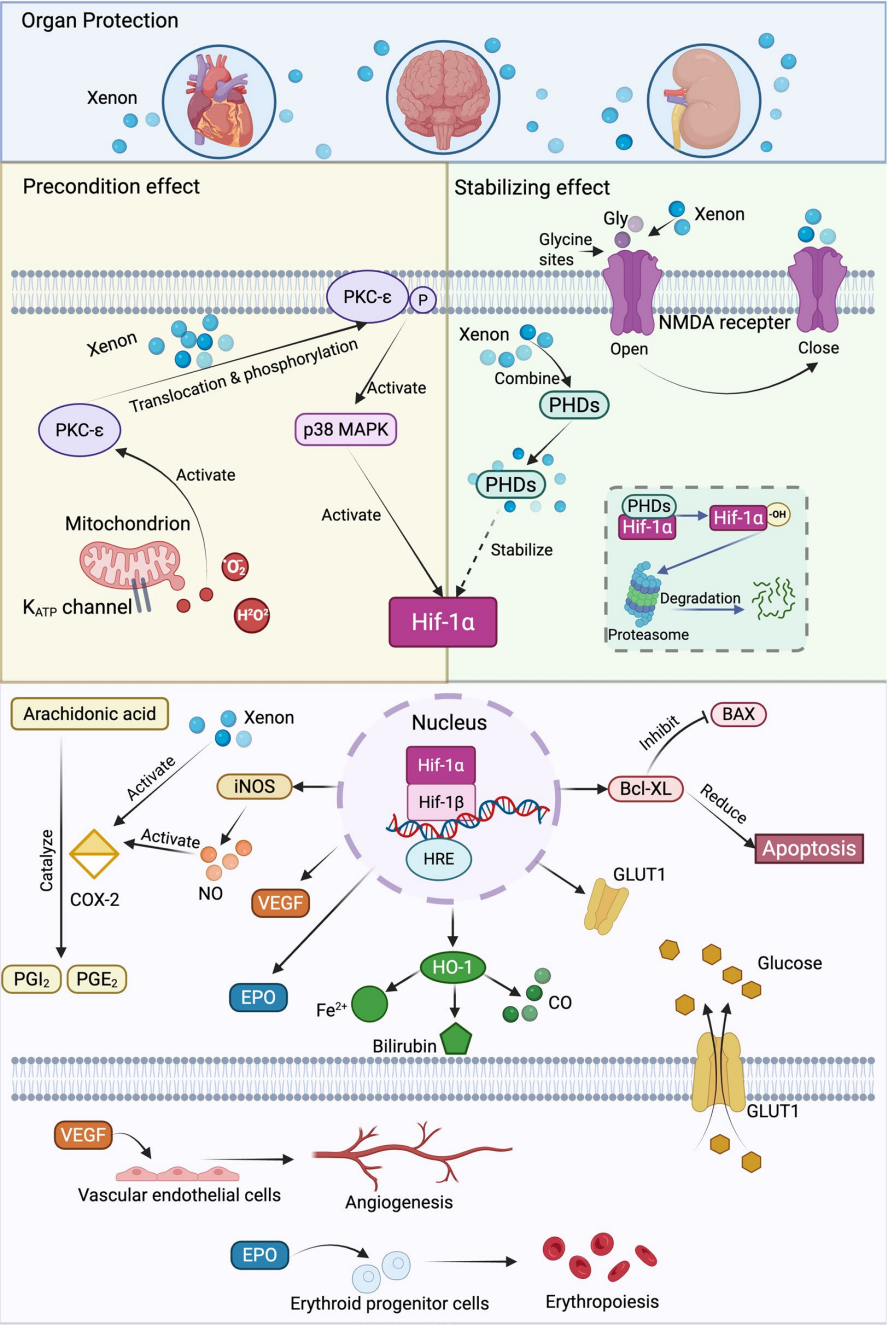
Xe modulates HIF-1 activation through multiple pathways. Xenon exhibits protective effects on the CNS, heart, and kidney. Xe exerts preconditioning effects through upstream regulators of HIF-1α, like PKC-ε and p38-MAPK, enhancing cellular resistance to ischemic injury. Xe binds to PHD, prolonging their transcriptional activity under hypoxic conditions. Xe can also competitively inhibit NMDA receptors by binding to glycine sites, reducing excitotoxicity and oxidative stress, which indirectly contributes to HIF-1 stabilization. In the nucleus, HIF-1α dimerizes with HIF-1β to bind HREs in target genes, thereby promoting the transcription of these genes and the expression of their encoded proteins. iNOS can produce NO, which activates COX-2 to promote arachidonic acid conversion into PGI2 and PGE2. Meanwhile, xenon can also enhance COX-2 activity, exerting cardioprotective effects. HO-1 generates Fe^2+^, bilirubin, and CO, maintaining cellular homeostasis by regulating oxidative stress and inflammatory responses. The increase in GLUT1 improves glucose uptake under hypoxic conditions, sustaining basal glucose metabolism. Bcl-XL and BAX are key genes in the apoptotic pathway, where Bcl-XL inhibits BAX to suppress apoptosis. HIF also increases the transcription of VEGF and EPO. The former binds to receptors on vascular endothelial cells to promote angiogenesis, while the latter binds to receptors on erythroid progenitor cells to facilitate erythropoiesis. CNS: central nervous system; HIF-1: hypoxia-inducible factor-1; PKC-ε: protein kinase C-epsilon; p38-MAPK: p38 mitogen-activated protein kinase; NMDA: N-Methyl-D-aspartic acid; HREs: hypoxia-responsive elements; iNOS: inducible nitric oxide synthase; NO: nitric oxide; COX-2: cyclooxygenase-2; HO-1: heme oxygenase-1; CO: carbon monoxide; GLUT1: glucose transporter 1; Bcl-XL: B-cell lymphoma-extra-large; BAX: Bcl-2-associated X protein; VEGF: vascular endothelial growth factor; EPO: erythropoietin

Xe modulates HIF-1 activation through multiple pathways (Fig. 1). One significant mechanism involves its pharmacological preconditioning effects *via* protein kinase C (PKC)-epsilon and p38 mitogen-activated protein kinase (MAPK), which are upstream regulators of HIF-1α stabilization. This preconditioning enhances cellular resistance to ischemic injury by upregulating genes involved in angiogenesis and metabolic adaptation [16]. Additionally, Xe competitively inhibits N-Methyl-D-aspartic acid (NMDA) receptors by binding to the glycine site, reducing excitotoxicity and oxidative stress. Since these processes can disrupt the cellular metabolic state, their suppression by Xe indirectly promotes a stable environment for HIF-1 stabilization [17]. Molecular docking studies suggest that Xe may also bind to proteins regulating HIF-1α stability, such as PHDs, potentially prolonging its transcriptional activity under hypoxic conditions [18]. Collectively, these interactions highlight the capacity of Xe to activate the HIF-1 system and enhance cellular responses to hypoxia. Furthermore, Xe serves as a potent HIF-1 activator by stabilizing HIF-1α, enhancing transcriptional activity, and promoting neuroprotection through VEGF and erythropoietin expression, which underlines its protective effects against ischemia-reperfusion injuries and hypoxic damage [19, 20].

Through the HIF-1 pathway, NMDA receptor inhibition by Xe mitigates excitotoxicity and oxidative stress, resulting in central nervous system (CNS) protection. In neonatal hypoxia-ischemia models, Xe combined with therapeutic hypothermia exhibits synergistic neuroprotection by enhancing HIF-1α stability, reducing infarction sizes, and improving long-term neurological outcomes. The TOBY-Xe trial explored the potential of Xe combined with moderate hypothermia in neonatal care [21]. Similarly, in traumatic brain injury (TBI) and subarachnoid hemorrhage models, Xe reduces neuronal loss, alters microglial activation patterns, and improves cognitive outcomes. These beneficial effects are consistent with HIF-1-driven cellular repair and neuroinflammatory modulation, suggesting a potential link [22, 23]. In addition to its neuroprotective properties, Xe exhibits cardiac and renal protective effects by activating HIF-1-mediated mechanisms. Animal studies showed that Xe induces late-phase cardiac preconditioning *via* cyclooxygenase-2 (COX-2) pathways, a downstream target of HIF-1. This preconditioning enhances myocardial resistance to ischemia-reperfusion injury [24]. Similarly, Xe post-conditioning has been shown to preserve graft function and reduce apoptosis in ischemia-reperfusion injury through activating HIF-1 pathways that mitigate oxidative stress and enhance tissue repair in porcine kidney models [25, 26].

Ar also provides significant neuroprotective and anti-ischemic effects. In TBI and ischemic models, Ar treatment reduced neuronal apoptosis, microglial activation, and inflammation, supporting its therapeutic potential in neurotrauma and ischemic events [27]. Similar to Xe, Ar has been found to augment hypothermic neuroprotection in neonatal asphyxia. This effect was associated with HIF-1α stabilization and modulation of survival pathways, suggesting its potential involvement in the underlying mechanism [28, 29]. While Xe demonstrates the most significant activation of the HIF-1 system, Ar exerts its effects through slightly different mechanisms, potentially interacting with protein-lipid interfaces to modulate hypoxia pathways, though its effects are less potent than those of Xe [30, 31]. Understanding the molecular interactions between noble gases and the cellular signaling system and their long-term effects on cellular adaptation to hypoxia will further expand therapeutic applications [32, 33].

### Suppression of regulated cell death

Cell death is a fundamental biological process essential for tissue homeostasis, but dysregulation exists under pathological conditions [34]. Various forms of regulated cell death are governed by distinct molecular pathways. Recent research highlights the potential of Xe and Ar in modulating these processes, offering significant therapeutic promise. Representative findings on negating regulated cell death of Xe and Ar were summarized in Table 1.

**Table 1:** Summary of xenon and argon effects on regulated cell death pathways

Regulated cell death	Gas	Mechanism	Model	References
**Apoptosis**	Xe	Prevent MMP and cytochrome C release and inhibit caspase activation	Human osteosarcoma cell line (U2OS)	[35]
		Reduce caspase-3 activation and preserve neuronal survival	Newborn piglet hypoxia-ischemia model	[36]
		Increase Bcl-2 expression, decrease cytochrome C release and P53 expression	Rat neuronal apoptosis model	[37]
		Suppress caspase-4 and NF-κB signaling pathway and increase IL-10 expression	Mouse septic acute kidney injury model	[38]
		Inhibit caspase-3 and protect neonatal neurons	Postnatal mice pups	[39]
	Ar	Reduce STAT3, NF-κB and FasL expression	Human neuroblastoma cell line	[40]
**Necroptosis**	Xe	Phosphorylate Akt and GSK-3β, preserve mitochondrial function, and inhibit Ca^2+^-induced mPTP opening	Rat LAD IRI model	[41]
**Ferroptosis**	Xe	Reduce iron accumulation, limit oxidative stress and suppress lipid peroxidation	Mouse neonatal hypoxic model	[42]
	Ar	Promote the translocation of TXNIP to mitochondria	Pig liver ischemia model	[43]
**Pyroptosis**	Xe	Downregulate renal NLRP3 inflammasome-mediated cellular signaling	Mouse lupus nephritis model	[44]
	Ar	Suppress microglial activation and reduce pyroptosis-associated inflammation	Rat retinal IRI model	[40]
**Autophagy**	Xe	Facilitate the clearance of damaged organelles and reduce oxidative stress	Rat seizure model	[45]
	Ar	Enhance autophagic flux, promote lysosomal function and reduce tissue inflammation	Rat stroke model	[46]

MMP: mitochondrial membrane permeabilization; Bcl: B-cell lymphoma; NF-κB: nuclear factor-kappa B; IL: interleukin; STAT3: signal transducer and activator of transcription 3; FasL: fas ligand; Akt: protein kinase B; GSK-3β: glycogen synthase kinase 3 beta; LAD: left anterior descending coronary artery; IRI: ischemia-reperfusion injury; TXNIP: thioredoxin-interacting protein; NLRP3: NOD-like receptor thermal protein domain-associated protein 3

#### Apoptosis: modulation of programmed cell death

Apoptosis is a tightly regulated process that eliminates damaged or unnecessary cells while maintaining tissue integrity. However, dysregulated or excessive apoptosis contributes to pathological tissue damage, as observed in ischemia or neurodegeneration [47]. Xe exerts anti-apoptotic effects across various preclinical models. Mechanistically, Xe stabilizes mitochondrial membranes, thereby preventing cytochrome c release and subsequent caspase activation [35]. In neonatal hypoxia-ischemia models, Xe significantly reduces caspase-3 activity and the expression of the pro-apoptotic tumor suppressor p53, while upregulating anti-apoptotic factors such as phosphorylated B-cell lymphoma (Bcl)-2, and brain-derived neurotrophic factor (BDNF), thus collectively preserving neuronal survival [15, 36, 37]. In addition, Xe preconditioning has been reported to reduce lipopolysaccharide (LPS)-induced renal apoptosis *via* upregulating microRNA-21, which in turn suppresses caspase-4 expression and nuclear factor (NF)-κB signaling, while increasing IL-10 production, thereby inhibiting inflammation and ultimately contributing to anti-apoptosis [38]. Interestingly, Xe may also exert context-dependent pro-apoptotic effects. In rodent models, Xe significantly reduces isoflurane-induced neuroapoptosis through inhibiting caspase-3 [39]; however, in a porcine model of myocardial infarction, Xe exposure during early ischemia-reperfusion induced greater apoptosis compared with isoflurane [48]. These findings indicate that while Xe generally exhibits cytoprotective properties, its effects may vary depending on the tissue type, timing, and pathophysiological context [49]. Similarly, Ar demonstrated anti-apoptotic activity in models of neuronal and retinal ischemia. Its mechanisms involve modulation of extracellular signal-regulated kinase (ERK1/2) signaling, TLR2/4, Nrf2, Bcl-2, and inhibiting caspase-9 activation. These actions contribute to the preservation of cellular viability and attenuation of apoptotic damage under ischemic stress [40, 50].

#### Necroptosis: mediation in ischemic injury

Necroptosis is a caspase-independent, inflammatory form of regulated cell death mediated by receptor-interacting protein kinase (RIPK1, RIPK3) and mixed lineage kinase domain-like pseudokinase (MLKL) [51]. This highly inflammatory process contributes to tissue damage in ischemic stroke and organ failure. Experimental study showed that Xe preconditioning downregulated necroptotic markers, reducing tissue injury in ischemia-reperfusion models [41].

#### Ferroptosis: protection against oxidation lipid peroxidation

Ferroptosis is driven by iron-dependent lipid peroxidation, leading to oxidative damage and cell membrane rupture. This form of cell death is particularly relevant in conditions involving neurodegenerative diseases and ischemic organ damage [52]. Xe attenuates ferroptosis by reducing iron accumulation and limiting oxidative stress. In neonatal hypoxia-induced seizure models, Xe suppressed lipid peroxidation, preserving neuronal viability [42]. Ar also counteracted ferroptosis by enhancing antioxidant defences, particularly by regulating thioredoxin-interacting protein (TXNIP), as observed in liver ischemia models [43].

#### Pyroptosis: modulation of inflammatory cell death

Pyroptosis is a highly inflammatory form of cell death driven by the NOD-like receptor thermal protein domain-associated protein 3 (NLRP3) inflammasome and caspase-1, leading to the release of pro-inflammatory cytokines, such as interleukin (IL)-1β and IL-18. This process exacerbated inflammation in conditions like ischemic stroke and autoimmune disorders [53]. Xe suppressed pyroptosis by inhibiting NLRP3 inflammasome activation, reducing inflammatory cytokine release, and improving outcomes in kidney disease models [44]. Ar also modulated inflammatory responses, suppressing microglial activation and reducing pyroptosis-associated inflammation in stroke models [40].

#### Autophagy: fine-tuning cellular homeostasis

Autophagy is a cellular process that degrades and recycles damaged organelles and proteins to maintain homeostasis. While generally protective, excessive or dysregulated autophagy can contribute to cell death under pathological conditions [54]. Xe has been shown to promote autophagy in kainic acid-induced seizure models, facilitating the clearance of damaged organelles and reducing oxidative stress [45]. Similarly, Ar enhanced autophagic flux in stroke models, promoting lysosomal function, reducing tissue inflammation and ultimately supporting tissue recovery [46].

Noble gases such as Xe and Ar demonstrate substantial potential in regulating various forms of cell death. By modulating apoptosis, necroptosis, ferroptosis, pyroptosis, and autophagy, these gases exhibit neuroprotective, anti-inflammatory, and cytoprotective properties. Future research should focus on elucidating the precise molecular mechanisms underlying noble gas-mediated cell death modulation and optimizing their clinical applications for neuroprotection and ischemic injury management.

### Inhibition of inflammation

Xe exhibits strong anti-inflammatory properties by targeting multiple pathways. One of its primary mechanisms involves the suppression of the NLRP3 inflammasome, a critical driver of innate immune responses. By inhibiting NLRP3 activation, Xe reduces the secretion of pro-inflammatory cytokines during lupus nephritis and sepsis [44]. Additionally, Xe enhanced tissue recovery by downregulating the production of tumor necrosis factor-alpha (TNF-α) and IL-6, which are central mediators of systemic inflammation [16, 36]. Another anti-inflammatory mechanism of Xe is the modulation of the mitogen-activated MAPK signaling cascade, thereby reducing oxidative stress and associated inflammatory damage [24].

Although less potent than Xe, Ar also exhibits notable anti-inflammatory properties. In models of neurological injury, Ar suppressed microglia activation, thereby reducing neuroinflammation and the release of pro-inflammatory cytokines. This effect was observed in stroke and traumatic brain injury, where Ar improved functional recovery and neuronal damage [40, 55, 56]. In liver ischemia-reperfusion models, Ar demonstrated protective effects by decreasing tissue inflammation and preserving function [43]. These findings suggest that Ar may serve as a viable therapeutic strategy for ischemia-reperfusion injury and inflammatory organ damage.

Both Xe and Ar share some overlapping mechanisms in their anti-inflammatory effects. They inhibit nuclear factor-kappa B (NF-κB), a transcription factor that drives the expression of inflammatory genes, leading to the reduced production of TNF-α, IL-6, and other inflammatory mediators [16, 57]. Additionally, both Xe and Ar prevent cell death processes, thereby diminishing the subsequent release of damage-associated molecular patterns (DAMPs) that amplify immune inflammation [44]. By inhibiting these processes, Xe and Ar help break the cycle of excessive immune activation and tissue damage. While these findings highlight the therapeutic potential of Xe and Ar, further research is needed to elucidate their precise molecular mechanisms and optimize their clinical applications. Large-scale clinical trials are essential to determine their safety, efficacy, and potential for long-term effects on human patients.

Although Ar and Xe may converge on some shared downstream transcriptional programs, their upstream potency and target engagement likely differ. Xe is heavier and more polarizable, enabling stronger non-covalent interactions within hydrophobic protein cavities and membrane-protein interfaces and yielding more significant functional modulation of receptor-level targets [58, 59]. In contrast, Ar interactions may be weaker and more context-dependent [60]. In addition, differences in achievable safe inspired fractions (while maintaining adequate FiO2), tissue partitioning, and heterogeneity in dose-time paradigms across Ar studies may contribute to more variable or apparently less potent anti-inflammatory effects compared with Xe [50, 61].

Moreover, Xe and Ar are promising anti-inflammatory agents with potential applications in conditions ranging from sepsis to neurodegenerative diseases. By targeting key inflammatory pathways such as the NLRP3 inflammasome, nuclear factor-kappa B (NF-κB) signaling, and inflammatory cell death mechanisms, they help reduce excess inflammation and promote tissue recovery. While Xe appears to have stronger anti-inflammatory effects, Ar also plays a valuable role, particularly in neurological and ischemic injuries. As research continues, clinical trials are crucial for determining the effective therapeutic integration of these gases. Their ability to modulate inflammation without significant toxicity makes them promising candidates for future therapies aimed at controlling immune responses and preventing inflammatory damage.

## Anesthetics and organ protection: latest advances and evidence

### Anesthesia of noble gases

The anesthetic properties of noble gases are influenced by their molecular interactions, primarily determined by van der Waal forces and solvent-effect energy. Anesthetic noble gases such as Xe exhibit a higher affinity for binding specific sites predominantly through van der Waal energy, while non-anesthetic noble gases are more reliant on solvent-effect energy [62, 63]. Structure analyses using molecular X-ray crystallography reveal that Ar binds to the outer hydrophobic surface of membrane proteins. Complementary molecular dynamics simulations have predicted potential binding sites within the lipid bilayer, suggesting a broader distribution of action [30]. The degree of noble gas solubility in lipid bilayers is largely determined by molecular size, which also influences their ability to modulate membrane fluidity. Noble gases are known to alter the lipid acyl chain order, counteracting the effects of elevated hydrostatic pressure and supporting the critical volume hypothesis in anesthesia pressure reversal [64].

Unlike traditional anesthetics that interact with gamma-aminobutyric (GABA) receptors, Xe exerts its effects predominantly through antagonism of N-methyl-D-aspartate (NMDA) receptors. This mechanism accounts for many pharmacological properties, including its analgesic, amnestic, neuroprotective, and anti-excitotoxic effects [65]. In addition, Xe also significantly inhibits the alpha-amino-3-hydroxy-5-methyl-4-isoxazole propionate (AMPA) and kainate receptor-mediated currents in cortical neurons, with a 40–66% reduction in AMPA/kainite activity and a 35–60% inhibition of GluR6-type kainate receptors [66]. These findings suggest that Xe exhibits a broad, non-selective inhibitory action across multiple glutamate receptor subtypes, which may enhance its clinical anesthetic and analgesic efficacy beyond pure NMDA antagonism.

Clinically, Xe is characterized by a minimum alveolar concentration (MAC) of 63% in humans. Its low blood-gas and brain-blood coefficients allow rapid induction and emergence from anesthesia, with little to no systemic toxicity and adverse hemodynamic effects [67–69]. Studies in both adults and children have confirmed that Xe maintains stable cardiovascular parameters and preserves organ oxygenation [70]. Large multicenter clinical trials have validated Xe as an effective and safe agent for general anesthesia and sedation in critically ill patients, offering the advantage of a more rapid recovery compared with conventional anesthetics [71, 72]. However, Xe may be related with a higher risk of postoperative nausea and vomiting in susceptible patients [73, 74]. Furthermore, compared to widely used inhalational anesthetic gases or propofol, Xe has been noted to lead to a higher mean arterial pressure, lower heart rate, and cardiac output [68, 75]. With regard to cerebral physiology, Xe increases white matter perfusion but reduces regional cerebral blood flow (rCBF) and glucose metabolism in specific grey matter regions, including the frontal cortex, thalamus, and cerebellum. These changes reflect known anesthetic-induced alterations in brain energetics and blood flow and suggest that Xe exerts typical anesthetic modulation of neurovascular dynamics [76–78].

Ar, although less studied, also demonstrates anesthetic properties under hyperbaric conditions. Emerging evidence suggests the anesthetic therapeutic effects of Ar, position it as a potential and inexpensive alternative to Xe [79, 80]. The mechanism by which Ar induces anesthesia is suggested to be *via* stimulation of γ-Aminobutyric acid sub-type A (GABAA) receptors. This finding, observed from rat experiments investigating the narcotic potency of Ar after pretreatment with GABA-receptor antagonists, showed a decreased narcotic potency of Ar following pretreatment with GABAA-receptor antagonists [81].

### Organoprotective effects of noble gases

Both Xe and Ar have demonstrated promising organoprotective effects across a range of injury models. The representative findings on the cytoprotective roles of Xe and Ar across organ systems are summarized in Tables 2 and 3.

**Table 2:** Recent evidence of xenon effects on Major organs

Organ	Main Findings	Molecular Mechanisms	Concentration	Model/Population	References
Brain	Protect dopamine neurons	Block NMDA glutamate receptor	25–75%	Midbrain culture	[82]
	Block propofol-induced neuronal damage/loss	Noncompetitive inhibit NMDARs	33%	Human-derived neural stem cell	[83]
	Induce metaphase arrest	Modulate Ca^2+^ influx	70%	Human cervical cancer cell line (HeLa S3) Human endothelial cell line (ECV 304)	[84]
	Reduce LDH release Reduce ischemic neuronal death in the cortex Decrease ischemic brain damage in the striatum	Decrease NMDA-induced Ca^2+^ influx Downregulate Bax Upregulate Bcl-xL Downregulate caspase-3 Active K^+^ ATP channels	70–75%	Mice neuron-glia co-culture Rat neuronal injury model	[28, 85, 86]
	Prevent HI neuronal damage	–	20–50%	Neonatal rat HI model	[87, 88]
	Reduce apoptosis/necroptosis in the brain	Inhibit RIPK-1, RIPK-3 and MLKL	70%	Rat exposed to sevoflurane Rat neonatal sepsis model	[19, 89]
	Improve the early neurological deficit and preserve neurological function	–	70%	Pig cardiac arrest model	[90, 91]
	Protect neuronal cells	Increase the gene transcription of ADNP	75%	Neonatal Sprague Dawley rat	[92]
	Reduce HI seizures, cognitive impairment, iron accumulation, and oxidative stress	Downregulate IRP1/DMT1 Upregulate FPN1	70%	Mice HI induced seizures model	[42]
	Anticonvulsant effect	–	30%	Infants with perinatal asphyxial encephalopathy	[93]
	Reduce the incidence of postoperative delirium	–	60%	Patients with hip fractures	[94]
	Reduce secondary neuronal injury post-insult	Inhibit NMDA receptors	75%	Mice hippocampal brain focal mechanical trauma models	[95]
	Improve neurological function, reduce contusion volume, prevent memory impairment, decrease neuroinflammation, reduce early locomotor deficits	–	75%	Mice/Rat controlled cortical impact model	[23, 32, 96]
	Improve social behavior, reduce exploratory drive, and normalize performance in the forced swim test	–	25%	Valproic acid-induced rodent model of autism	[97]
	Normalize corticostriatal synaptic dysfunction and reverse maladaptive plasticity Reduce dyskinetic movements and improve gait deficits	Inhibit NMDA receptors and reduce NMDA/AMPA rate	50%	Rat and macaque parkinsonism and dyskinesia model	[98]
	Attenuate medial hippocampus damage and reduce microglial activation	–	50%	Rat SAH model	[22]
	Reduce initial injury and prevent subsequent injury development	–	50%	Mice TBI model	[99]
	Protect DA neurons that are vulnerable in Parkinson’s disease	Inhibit NMDA receptors and glial cell proliferation, andmodulate intracellular calcium homeostasis	75%	Rat midbrain culture	[100]
	Promote a pre-neurodegenerative microglia state in microglia and suppress the microglial proinflammation	Enhance IFN-γ signaling pathway	30%	Mouse AD model	[101]
	Reduce fear memory in rats	–	25%	Rat PTSD model	[102]
**Heart**	No significant observations	–	60%	Patients without cardiac diseases scheduled for elective surgery	[103]
	Reduce infarct size	Active PKC-ε and downstream p38 MAPK	70%	Rat coronary artery occlusion model	[16, 24]
	Improve cell survival	Increase HIF-1α and VEGF	70%	Rat primary cardiac ventricular myocyte hypoxia model	[104]
	Preserve mitochondrial integrity, thereby protecting cardiac function	Activate mitochondrial ATP-sensitive potassium (mitoK ATP) channels	35%	Rabbit heart IRI model	[105]
	Trigger pro-inflammatory responses and suppress anti-inflammatory pathways	IL-6 increase IL-10 decrease	45–50%	Patients underwent coronary artery bypass grafting surgery	[106, 107]
	Reduce troponin-T release	–	40%	Out-of-hospital cardiac arrest patients	[108, 109]
**Lung**	Mitigate lung injury after kidney transplantation	Active PI3K/Akt/mTOR pathway, attenuate HIF-1α and HMGB-1, active TLR-4 and NF-κB, increase p-mTOR and Bcl-2.	70%	Human lung epithelial cell (A549) Rat kidney transplant model	[110]
	Increase airway resistance and peak inspiratory pressure	–	60%	Patients without pulmonary disease	[111]
	Reduce pulmonary inflammation	–	30%	Mice acute respiratory distress syndrome model	[112]
	No Significant observations	–	70%	Pig warm ischemic lung injury model	[113]
	Lung function heterogeneous abnormalities	–	87%	Interstitial lung disease patient	[114]
**Kidney**	No significant observations	–	70%	Pig kidney transplantation model	[25]
	Suppress T-cell infiltration and fibrosis Enhance cell proliferation provide morphologic and functional renoprotection	Increase IGF-1 and IGF-1 R via mTOR and HIF-1α activation	70%	Human proximal tubular HK-2 cells Rat kidney transplant model	[18, 26]
	Reduce cell death and inflammation	Bcl-2, p-Akt and HIF-1α expression upregulation	70%	Human proximal tubular HK-2 cells	[115]
	Suppress renal tubular damage, apoptosis, and LPS-induced inflammation	Upregulate miR-21 antiapoptotic factor. Inhibit PTEN/Akt proapoptotic signaling TNF-α, IL-6 blockage, and IL-10 elevation.	60%	Mice LPS-Induced AKI model	[38]
	Reduce proinflammatory cytokines and ROS	Decrease IL-1β, TNF-α, IL-15 and IL-6. Deactive NF-κB and NLRP3. Increase HIF-1α.	70%	Mice lupus nephritis model	[44]
	Reduce adverse events	–	60%	Patients with regular renal function undergoing partial nephrectomy.	[116]
**Liver**	Reduce portal venous flow and influence hepatic tissue oxygenation	–	65%	Anesthetized Pigs	[117]
	Protective effects against apoptosis, necroptosis, and ferroptosis in cold-preserved porcine livers	–	50%	Porcine model, cold-preserved livers	[43]

NMDA: N-Methyl-D-aspartic acid; HeLa S3: Human cervical cancer cell line; ECV 304: Human endothelial cell line; RIP: Receptor-Interacting Protein Kinase; MLKL: Mixed Lineage Kinase Domain-Like Protein; LDH: lactate dehydrogenase; Bax: Bcl-2-associated X protein; Bcl-xL: B-cell lymphoma-extra large; ATP: adenosine triphosphate; HI: hypoxic/ischemic; ADNP: activity-dependent neuroprotective protein; RIPK: receptor-interacting protein kinase; IRP1: iron regulator protein 1; DMT1: divalent metal transporter 1; FRN1: Ferroportin 1; SAH: subarachnoid hemorrhage; TBI: traumatic brain injury; IFN-γ: interferon-gamma; AD: Alzheimer’s disease; PKC-ε: protein kinase C-epsilon; mitoK ATP: ATP-sensitive potassium; DA: dopamine; PTSD: post-traumatic stress disorder; MAPK: mitogen-activated protein kinase; HIF-1α: hypoxia-inducible factor-1 alpha; VEGF: vascular endothelial growth factor; IRI: ischemia-reperfusion injury; IL: interleukin; PI3K: phosphoinositide 3-kinase; Akt: protein kinase B; mTOR: mechanistic Target Of Rapamycin; HMGB-1: high mobility group box-1; TLR: toll-like receptor; NF-κB: nuclear factor-kappa B; Bcl-2: B-cell lymphoma 2; IGF-1: insulin-like growth factor-1; HK-2: human kidney-2 proximal tubular cell line; LPS: lipopolysaccharide; PTEN: phosphatase and tensin homologue; TNF-α: tumor necrosis factor-alpha; AKI: acute kidney injury; ROS: reactive oxygen species; NLRP3: NOD-like receptor thermal protein domain-associated protein 3

**Table 3:** Recent evidence of Argon effects on Major organs

Organ	Main Findings	Molecular Mechanisms	Concentrations	Model/Population	References
**Brain**	Improve sensorimotor function, reduce brain edema and neuroinflammation	Reduce microglial/macrophage activation; upregulate microglia/macrophage polarisation to anti-inflammatory M2 phenotypes	50–75%	Mice traumatic brain injury model	[27, 40, 46, 79]
	Improve EEG activity recovery, brain energy metabolism and reduce cell death	–	45–50%	Pig perinatal asphyxia model	[29]
	Improve neuronal survival, neurological outcomes, and reduce infarct volumes	Upregulation of Bcl-2 and Bcl-xL; inhibition of excessive Bax expression	70%	Rat neonatal asphyxia model	[28]
	Decrease infarct size and hippocampal astrocyte activation.	Upregulate p-Akt and HO-1, inhibite p-GSK-3β Tyr-216, and suppressed NF-κB activation	70%	Rat hypoxic–ischemic brain injury model	[118]
	Block amphetamine-induced locomotor sensitization	Inhibit VMAT2 and antagonize μ-opioid receptors	75%	Rat amphetamine-induced locomotor sensitization model	[119]
	Promote neurological recovery, reduce cortical neuronal degeneration, inhibit hippocampal microglia activation and decrease circulating biomarkers of brain injury	–	70%	Pig cardiac arrest model	[120]
	Protect neuronal cells and reduce apoptosis	Decrease the expression of TLR2, TLR4, Bax, caspase-3, IL8, HSP70 and HO-1; activate ERK1/2, inhibit NF-κB phosphorylation and suppress Akt phosphorylation	74%	Human neuroblastoma cell line (SH-SY5Y)	[50]
	Reduce neuronal damage after cardiac arrest	–	70%	Rat cardiac arrest model	[121]
**Heart**	Reduce apoptosis	Active Akt, ERK and biphasic JNK	50%	Rat cardiac arrest model	[122]
	Improve stroke volume, cardiac output and coronary flow	Increase JNK	50%	Rat heart IRI model	[123]
	Reduce infarct size of left ventricular area	Activate prosurvival kinases and inhibit MPTP opening	30%	Rabbit left anterior descending coronary artery occlusion and reperfusion model	[124]
	Eliminate arrhythmias, prevent the decrease in left ventricular ejection fraction and increase in wall motion	Inhibit MPTP and active RISK pathway	80%	Pig guinea ventricle model and rat myocardial IRI model	[125]
**Lung**	Restore lung architecture	IL-1β, IL-4 and LIF downregulated	70%	Mouse neonatal sepsis model	[126]
**Liver**	Reduce cell death and oxidative stress; increase plasma membrane integrity	Promote the translocation of TXNIP to mitochondria	50%	Porcine model, cold-preserved livers	[43]
	Reduce apoptosis	Inhibit caspase-3 activation	75%	Human osteosarcoma cell line (U2OS)	[35]
	Stimulate the antioxidant system; upregulate free radical oxidation	–	–	Wistar rat	[127]
	Inhibit the regeneration of the liver	Increase IL-β and IL-6, decrease Ki-67	50%	Rat liver IRI model	[128]
	Reduce hepatocyte proliferation potential	Decrease IL-6	50%	Rat partial hepatectomy model	[129]
**Kidney**	Improve pig survival and renal function recovery after transplantation	Alter antioxidant defences and reduce inflammation response	100%	Pig kidney transplant model	[130]

EEG: electroencephalogram; Bcl-2: B-cell lymphoma 2; Bcl-xL: B-cell lymphoma-extra large; Akt: protein kinase B; HO-1: heme oxygenase-1; p-GSK-3β : phosphorylated glycogen synthase kinase-3 beta; NF-κB: nuclear factor-kappa B; VMAT2: vesicular monoamine transporter 2; TLR: toll-like receptor; Bax: Bcl-2-associated X protein; IL: interleukin; HSP70: heat shock protein 70; ERK: extracellular signal-regulated kinase; JNK: c-Jun N-terminal kinase; IRI: ischemia-reperfusion injury; MPTP: mitochondrial permeability transition pore; RISK: reperfusion injury salvage kinase; LIF: leukemia inhibitory factor; TXNIP: thioredoxin-interacting protein

### Xenon

#### Brain

The neuroprotective effects of Xe are primarily mediated through antagonism of the NMDA subtype of the glutamate receptor, thereby mitigating neuronal excitotoxicity [82]. In a mouse neuron–glia co-culture system, Xe reduced neuronal injury in a concentration-dependent manner, as reflected by decreased lactate dehydrogenase release following exposure to NMDA, glutamate, or oxygen deprivation [131]. Additionally, Xe reduced propofol-induced neurotoxicity in human neural stem cell-derived neuronal cultures [83]. In both in vivo middle cerebral artery occlusion (MCAO) rat models and in vitro hypoxic cortical neuron models, Xe attenuated excitotoxic neuronal death *via* modulation of Ca^2 +^ influx, a central mechanism in neuronal injury [20, 84, 132]. The combination of Xe with therapeutic hypothermia has demonstrated synergistic neuroprotection in neonatal asphyxia models, evidenced by reduced infarct volume, improved neurological function, and enhanced histopathological profiles [87, 88]. Mechanistically, this effect involves upregulation of anti-apoptotic Bcl-xL, downregulation of pro-apoptotic BCL-2-associated X (Bax), and inhibition of caspase-3 activation [19, 28, 85]. In models of cardiac arrest, administration of 70% Xe in combination with mild hypothermia (33°C) preserved neurological function, reduced perivascular inflammation, and limited neuronal necrosis in the putamen [90, 91]. Xe preconditioning has also been shown to increase the gene transcription of activity-dependent neuroprotective protein (ADNP) [92] and activate recombinant ATP-sensitive potassium channels, emulating the neuroprotective mechanisms [86]. It was also reported that Xe protected against neuronal necroptosis induced lipopolysaccharide in rat neonates and subsequently improved cognition in adulthood [89].

Clinically, inhalation of 30% Xe for 24 hours combined with 72 hours of moderate systemic hypothermia elicited anticonvulsant effect in neonates with perinatal asphyxia [93]. In elderly patients, Xe anesthesia has been associated with reduced postoperative delirium [94], although some trials have reported no significant difference in neurological outcomes following cardiac surgery [133]. While Xe inhalation reduced ischemic white matter injury in one study, no significant differences in long-term mortality or neurological outcomes were observed at 6 months [134]. Meta-analyses have highlighted the superiority of Xe over traditional anesthetics like propofol in maintaining oxygen homeostasis, supporting its broader adoption in clinical practice [135].

Xe has also shown promising efficacy in various models of TBI. In hippocampal brain slice models subjected to focal mechanical trauma, Xe reduced secondary neuronal injury post-insult [95]. This effect was reversed by increased glycine concentrations, indicating Xe involves inhibition at the NMDA receptor glycine-binding site [136]. In TBI mouse models, 30 and 50% Xe treatment led to improved neurological function and significantly reduced contusion volume [32]. In rat models of subarachnoid hemorrhage (SAH), 50% Xe markedly attenuated damage in the medial hippocampus and reduced microglial activation in the piriform cortex [22]. In a novel blast-TBI model induced by single shockwave exposure, Xe not only mitigated initial injury but also halted the progression of secondary damage [99]. Furthermore, Xe treatment prevented late-onset TBI-associated memory impairment, with long-term neuroprotection attributed to decreased neuroinflammation [23]. In controlled cortical impact models, Xe improved locomotor function and promoted early-phase beneficial neuroinflammatory responses [96].

Xe has shown potential in modulating neurodevelopmental and neurodegenerative conditions. In valproic acid (VPA)-induced autism mice, Xe improved the balance between excitatory and inhibitory neurotransmission in the CNS, leading to enhanced social interactions, reduced exploratory activity, and normalized performance in the forced swim test [97]. In animal models of levodopa-induced dyskinesia, Xe reversed corticostriatal synaptic dysfunction and reduced dyskinesia neuroplasticity, while reducing dyskinetic movements and improving gait deficits in both rats and non-human primates [98]. Through directly antagonizing NMDA receptors and indirectly by suppressing glial cell-mediated toxicity while supporting neurotrophic mechanisms, Xe may protect dopaminergic neurons, suggesting a novel therapeutic application for Parkinson’s disease [100]. Moreover, recent research has demonstrated that Xe inhalation promotes a pre-neurodegenerative microglia state in microglia through interferon (IFN)-γ signaling, indicating the potential usage of Xe as a therapeutic strategy for Alzheimer’s disease [101]. Further research has investigated the use of Xe in targeted drug delivery systems. Xe-containing echogenic liposomes (Xe-ELIP), activated with ultrasound, demonstrated a controlled two-phase release in subarachnoid hemorrhage. Xe-ELIP significantly reduced cerebral bleeding, improved neurological outcomes, and decreased neuronal apoptosis, suggesting the potential of noninvasive, ultrasound-triggered Xe delivery strategies [137]. Finally, Xe has shown promise in modulating traumatic memory. Inhalation of 25% Xe for 1 hour immediately following memory reactivation significantly attenuated fear memory in rats, with effects lasting up to 18 days. These findings suggest Xe may represent a viable therapeutic candidate for post-traumatic stress disorder (PTSD) and related anxiety disorders [102].

#### Heart

Xe is widely recognized for its cardioprotective properties, particularly in the context of ischemia-reperfusion injury. Preconditioning with Xe has been shown to reduce infarct size following myocardial ischemia in various animal models [16, 138, 139]. In rat hearts, a brief 5-minute preconditioning with Xe significantly reduced infarct size *via* activation of PKC-ε and its downstream target p38 MAPK [16, 140]. Other studies have demonstrated that Xe preconditioning upregulates HIF-1α and vascular endothelial growth factor (VEGF) expression, although not COX-2. However, inhibition of COX-2 abolished cardioprotective effects of Xe, suggesting its indirect involvement [24, 104]. In immature rabbit hearts, 75% Xe activated mitochondrial ATP-sensitive potassium channels and preserved mitochondrial integrity, thereby protecting cardiac function during ischemia-reperfusion [105]. More recently, oral administration of Xe-enriched solutions in ApoE^−^/^−^ mice maintained blood pressure and preserved left ventricular structure [138].

Clinical studies found the feasibility and safety of Xe [106], with one series demonstrating that Xe combined with therapeutic hypothermia reduced troponin-T release and myocardial injury in patients after out-of-hospital cardiac arrest [108, 109]. Similarly, a multicenter randomized trial reported superior cardiovascular stability with xenon compared to isoflurane in the same patient population [103]. In addition, Xe anesthesia preserves left ventricular function during surgery and minimizes hemodynamic instability, making it a safer anesthetic for high-risk cardiac surgical patients [106]. However, contrasting findings have also emerged, with one study indicating that Xe may trigger pro-inflammatory responses and suppress anti-inflammatory pathways in patients undergoing on-pump coronary artery bypass grafting [107].

#### Lung

Studies investigating the effects of Xe on the lungs are limited. A previous study found that post-conditioning with Xe protected the lung against remote injury following kidney transplantation in rats [110]. Xe treatment activated the PI3K/Akt/mTOR pathway in the lung epithelium. This led to elevated production of the protective factor HIF-1α, which in turn attenuated High mobility group box (HMGB)-1 translocation and Toll-like receptor (TLR)-4 and NF-κB activation. However, blocking the m-TOR-HIF-1α using HIF-1α siRNA blocked the Xe-mediated pulmonary protection. In patients with healthy lungs, Xe administration led to increased airway resistance and peak inspiratory pressure. This suggests a potential decreasing effect of Xe on the bronchial diameter, which may aggravate airway obstruction [111]. In acute respiratory distress syndromes, Xe inhalation reduced the inflammatory process in lung tissues, as reflected by decreased lung and body weight [112]. However, in pig models of warm ischemic lung injury, no difference was found in lung function during prolonged ex vivo lung perfusion using Xe or Ar [113]. Hyperpolozied Xe (129Xe) has also enabled the detection of highly heterogeneous abnormalities in lung function not captured through standard pulmonary function tests in patients with interstitial pneumonia [114]. Although these studies indicate an organoprotective effect of Xe on the lungs, further validation is necessary.

#### Kidney

In rat kidney allografts, Xe exposure before and after transplantation enhanced insulin-like growth factor 1 receptor (IGF-1 R) expression and reduced T-cell infiltration and renal fibrosis [26]. Similarly, Xe pretreatment in hypoxia-ischemia injury human kidney (HK)-2 cells reduced cell death and inflammation, while promoting increased cell proliferation via IGF-1-receptor, mTOR, and HIF-1α, as well as enhanced p-Akt and Bcl-2 expression [115]. Furthermore, Xe preconditioning was found to be renoprotective against ischemia-reperfusion injury in mice. Xe suppressed renal tubular damage, apoptosis and Lipopolysaccharides (LPS)-induced inflammation. miR-21 anti-apoptotic factor was also upregulated in the kidney, while Phosphatase and tensin homologue (PTEN)/Akt proapoptotic signaling was inhibited. LPS-induced production of TNF-α and IL-6 was blocked, and anti-inflammatory factor IL-10 was elevated [38]. The protective effects of Xe may occur through enhancing HIF-1α expression via mTOR pathways [18]. Moreover, an elegant study also demonstrated that 70% Xe lowered pro-inflammatory serum cytokines, including IL-1β, TNF-α, IL-15, IL-6, and reactive oxygen species (ROS) production, suppressed NF-κB/NLRP3 inflammasome activation and attenuated severe lupus nephritis in a pre-clinical model [44]. Xe also inhibited NF-κB and NLRP3 activation, key regulators of the inflammatory response. In humans, Xe administration was found to be safe and feasible in patients with partial nephrectomy. A previous study [116] reported reduced adverse events in the Xe-treated group compared to isoflurane and no changes to postoperative glomerular filtration rate and, hence, kidney function.

#### Liver

In two porcine model studies, Xe was shown to alter hepatic oxygenation, evidenced by increased oxygen content in hepatic venous blood without changes in hepatic perfusion distribution in one study [141], and by the absence of an expected increase in liver surface PO₂ during systemic hyperoxia in another [117]. These findings may be attributed to reduced plasma catecholamine levels or microvascular redistribution within the liver. Additionally, Xe, in combination with dexmedetomidine, has demonstrated protective effects against apoptosis, necroptosis, and ferroptosis in cold-preserved porcine livers [43].

### Argon

#### Brain

Ar was demonstrated to be neuroprotective in various brain injury models. In mice, administration of 70% inhaled Ar following TBI led to improved sensorimotor function and reduced brain edema [79]. Comparable neuroprotective effects were observed in in vitro models, including oxygen-glucose deprivation and TBI in organotypic hippocampal slice cultures from mice [142, 143]. Mechanistically, Ar has been shown to downregulate neuroinflammatory responses *via* reduced microglial/macrophage activation [27, 40, 79], and upregulated polarization towards the anti-inflammatory M2 phenotypes [46, 144]. Although these immunomodulatory effects may attenuate secondary injury development, some studies report no significant reduction in primary trauma severity when compared to the standard atmosphere [145]. In perinatal asphyxia pig models, treatment of 45–50% Ar during cooling at 33 °C from 2 to 26 hours post-injury, improved electroencephalogram (EEG) activity recovery, brain energy metabolism and reduced cell death [29]. Studies also highlight markedly improved neuronal survival, neurological outcomes, and reduced infarct volumes [28, 46, 146]. These benefits have been linked to activation of neuroprotective signaling pathways such as Heme Oxygenase-1 pathway [118] and ERK 1/2 signaling in neurons and microglia [147]. Interestingly, Ar may also have potential applications in treating substance-use disorders. Preclinical data suggest that Ar reduce drug-induced locomotor sensitization by antagonizing vesicular monoamine transporter-2 (VMAT2) and μ-opioid receptors in the nucleus accumbens, indicating a possible role in modulating addiction-related behaviors [119].

However, evidence varies in the neurological outcomes following cardiac arrest. When ventilated with 70% Ar, pig models showed improved neurological recovery and reduced cortical neuronal degeneration, hippocampal microglia activation and circulating biomarkers of brain injury [120]. In rat models, 70% of Ar administered post-resuscitation led to improved neurological function and reduced neocortical and hippocampal neuronal damage. Evidence suggests that the neuroprotective effect of Ar is dose-dependent [125], with a majority of studies using 70% Ar, and is not mediated by ATP-dependent potassium channels [121]. Conversely, in a mouse model of cold-head injury (CHI), inhalation of 70% or 79% Ar for 24 hours did not produce significant improvements in neurological outcome, nor did it affect markers of neurodegeneration or neuroinflammation. These contrasting findings underscore the importance of rigorous preclinical evaluation to delineate the specific contexts in which Ar confers neuroprotection [148].

#### Heart

Ar exhibits cardioprotective effects. Pre-conditioning cardiac myocyte-like progenitor cells with 30 and 50% Ar before oxygen-glucose deprivation, decreased both early and late apoptosis *via* Akt, ERK and biphasic c-Jun N-terminal kinase (JNK) activation [122]. In a rabbit model of left anterior descending coronary artery occlusion and reperfusion, 70% Ar significantly reduced infarct size by activating pro-survival kinases and inhibiting mitochondrial permeability transition pore (mPTP) opening [124]. Similarly, 50% Ar preconditioning in ischemia-reperfusion injury rat models reduced JNK activation with improved stroke volume, cardiac output and coronary flow [123]. Ar post-conditioning also eliminated arrhythmogenic events and prevented reductions in left ventricular ejection fraction and increases in wall motion abnormalities [125]. However, not all studies report beneficial effects. In one rat model, inhalation of 50% Ar for 24 hours post-resuscitation from cardiac arrest did not improve either histological damage or clinical outcomes [149], indicating that while Ar holds promise as cardioprotective agent, its efficacy may be context-dependent and requires further investigation.

#### Lung

Evidence varies regarding the effect of Ar on the lungs. In a neonatal mouse model, exposure of 70% of Ar post-sepsis induction restored lung architecture, improved survival and downregulated IL-1β, IL-4 and Leukemia inhibitory factor (LIF) [126]. However, in porcine models, Ar exposure before, during and after ischemia-reperfusion injury did not improve pulmonary graft function or physiological parameters [150].

#### Kidney

Ar saturation in Celsior solution significantly improved pig survival and renal function recovery after kidney transplantation, which may be mediated by altering antioxidant defending mechanisms and inflammation responses [130]. However, Evidence showed that inhalation with Ar post kidney transplantation did not improve graft function in pigs [25].

#### Liver

50% Ar may modulate oxidative stress and apoptosis in the liver. Cold-preserved pig liver grafts with 0.1 nM dexmedetomidine and 50% Ar showed reduced apoptosis, necroptosis and ferroptosis and oxidative stress and increased plasma membrane integrity [43]. Ar may exhibit these antiapoptotic effects through inhibiting caspase-3 activation. Furthermore, a previous study [128] showed upregulated IL-β and IL-6 expression and decreased Ki-67, indicating detrimental effects on liver regeneration. On the other hand, another study [129] demonstrated reduced IL-6 and hepatocyte proliferation potential following Ar application.

## Administration, dosing, delivery systems, and regulatory considerations

Xe and Ar are most commonly administered as inhaled medical gases *via* mask, ventilators or anesthesia machines. This delivery approach is analogous in principle to therapeutic oxygen administration. Dosing is defined by the inspired gas fraction (FiXe or FiAr), exposure duration, and timing of the therapeutic window at pre-, peri-, and post-conditioning manners. It is complemented by a continuous certain concentration of oxygen supply (e.g. more than 30%) under inspired and end-tidal gas concentration monitoring together with pulse oximetry monitoring as well [151–153]. Although clinical translation of Ar remains limited by the lack of standardized human dosing regimens and efficacy trials, the concentration ranges used in preclinical studies for both gases are generally similar, often 25–70% (better less than 50%) balanced with oxygen and/or nitrogen [50, 120, 154, 155]. Beyond inhalation, both gases have been explored preclinically through other exposure modalities, such as saturation in preservation solutions or incorporation into ex vivo perfusion circuits [43, 130, 156, 157]. A key practical distinction is that Xe often requires closed- or semi-closed circuits with recycling capability to reduce consumption and enable cost-effective administration [154, 158]. In contrast, Ar is generally delivered *via* conventional gas blending equipment, provided oxygen-displacement risks are managed by maintaining adequate FiO2 and appropriate alarm limits [153]. From a regulatory and quality perspective, Xe and Ar are primarily obtained *via* industrial air separation processes. Xe is a rare by-product and requires additional purification steps, which contribute to cost and supply constraints. Therapeutic use of both gases requires medicinal- or medical-grade gases manufactured under GMP/cGMP standards, which differ from industrial-grade gases used in non-medical applications [159, 160]. Xenon has already obtained clinic market authorization in parts of Europe [161, 162]. In clinical practice, Xe and Ar are administered as oxygen-containing mixtures, not as pure agents. However, for Ar, key feasibility barriers remain, including heterogeneity in preclinical models and endpoints, uncertainty regarding optimal dose-time profiles, and the need for standardized delivery and monitoring protocols. The primary safety concern for both gases is the risk of hypoxia or asphyxiation risk due to oxygen displacement. This underscores the necessity of regulated delivery infrastructure, reliable gas analytics, and appropriate clinical governance. The translational comparison of Xe versus Ar showed in Table 4.

**Table 4:** Translational comparison of Xe versus Ar

	Xenon (Xe)	Argon (Ar)	References
**Cost/supply**	High cost, limited supply; Cost is a major barrier to routine clinical use.	Low cost, abundant supply; Generally easier to source at scale.	[158]
**Delivery complexity**	Higher complexity; Often requires closed/semi-closed circuits and recycling to reduce consumption; Specialized anesthesia/ventilation solutions may be needed for practical implementation.	Lower complexity; Typically feasible with standard ventilators and anesthesia machines plus gas blending, provided safe FiO₂ is maintained; Does not inherently require recycling infrastructure.	[153, 154]
**Monitoring requirements**	More stringent and technology-dependent; Continuous monitoring of oxygenation plus end-tidal and inspired Xe concentration and gas analysis integrated into delivery systems; Tight control to ensure target exposure while managing consumption.	Standard critical-care level monitoring is usually sufficient; Continuous oxygenation monitoring is essential; Gas analysis for FiAr is beneficial but often less specialized than Xe platforms; Main safety focus is preventing hypoxic mixtures due to oxygen displacement.	[153, 158–160]
**Regulatory maturity**	More mature; Has established history as an anesthetic agent in parts of Europe.	Less mature for therapeutic indications; Predominantly investigational for organ protection and anti-inflammatory applications; Fewer formal clinical pathways and less consensus on standardized dosing, delivery, and endpoints.	[161, 162]

## Conclusion, challenges, and future directions

**Fig. 2:**
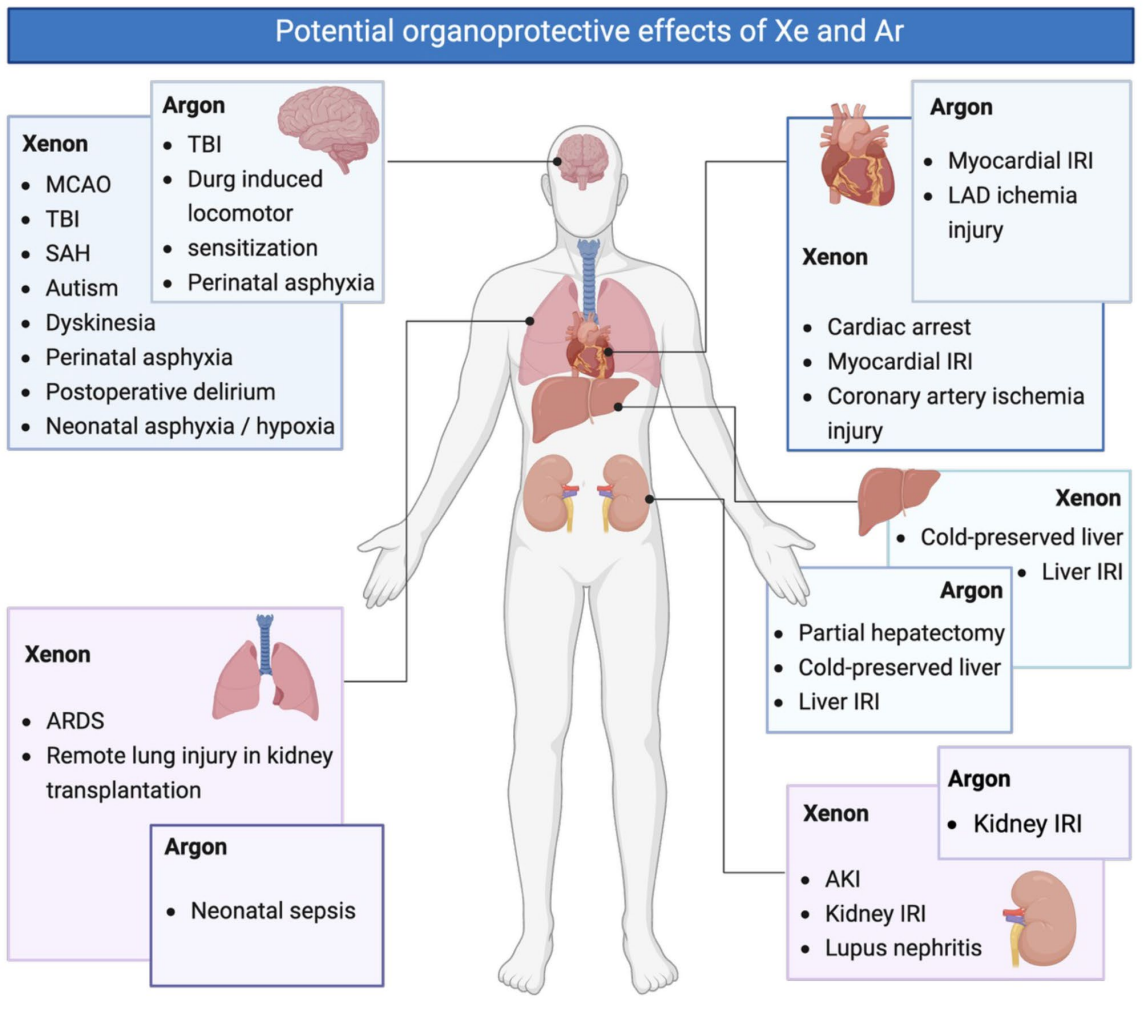
Potential organoprotective effects of xenon and argon. The protective effects of xenon on perinatal asphyxia, postoperative delirium, preservation of cognitive function, and cardiac arrest have been clinically established, additional therapeutic benefits of Xe and Ar on heart, lung, liver, and kidney observed in preclinical studies warrant further validation. MCAO: middle cerebral artery occlusion; TBI: traumatic brain injury; SAH: subarachnoid hemorrhage; ARDS: acute respiratory distress syndrome; IRI: ischemia-reperfusion injury; LAD: left anterior descending coronary artery; AKI: acute kidney injury

Noble gases Xe and Ar have demonstrated substantial promising organoprotection. The protective effects of Xe in patients, particularly in perinatal asphyxia, postoperative delirium and cardiac arrest, have been clinically validated [93, 94, 108, 109]. Meanwhile, additional beneficial effects of both Xe and Ar, demonstrated in animal models, require further investigation (Fig. 2). The unique physical and chemical properties of Xe and Ar enable them to modulate crucial molecular pathways associated with HIF-1, regulated cell death, and inflammation, making Xe and Ar valuable therapeutic agents in managing ischemia-reperfusion injury, traumatic brain injury, and systemic inflammation across a variety of organs.

However, several challenges remain to be tackled. Xe is limited by high cost and availability, and the need for specialized delivery systems [158, 163]. Ar, while more abundant and affordable, exhibits inconsistent efficacy across experimental models, with variable outcomes, particularly in liver [127, 128] and lung [150] protection. Additionally, many of the proposed mechanisms remain insufficiently understood, and the translation of preclinical findings into clinical applications is still in its early stages.

Future research should prioritize mechanistic clarity, including the identification of specific gas-protein and gas-lipid interactions that mediate biological effects [164]. Large-scale, well-designed clinical trials are needed to evaluate safety, efficacy, and long-term outcomes, particularly for Ar, which lacks comprehensive human data. The development of cost-effective delivery and recycling systems for noble gases will be critical to their wider adoption in clinical practice. Moreover, exploring combination strategies, such as combining noble gases with hypothermia or pharmacologic drugs, may yield synergistic benefits in complex diseases. Moreover, the identification of biomarkers that predict therapeutic responses likely further enhances outcomes in certain high-risk patient populations.

In summary, the anesthetic and organoprotective roles of Xe and Ar represent a novel and exciting frontier in translational medicine. While Xe already shows strong clinical potential, Ar offers a more accessible alternative that needs deeper investigation. Addressing the current limitations through coordinated scientific and clinical efforts will be key to exploring the full therapeutic value of noble gases in modern medicine.

## Data Availability

Not applicable.

## Abbreviations

Xe: Xenon
Ar: Argon
HIF-1: Hypoxia-inducible factor-1
PHDs: Prolyl hydroxylase domains
HREs: Hypoxia-responsive elements
VEGF: Vascular endothelial growth factor
PKC: Protein kinase C
MAPK: Mitogen-activated protein kinase
NMDA: N-Methyl-D-aspartic acid
CNS: Central nervous system
TBI: Traumatic brain injury
COX-2: Cycloocygenase-2
Bcl: B-cell lymphoma
BDNF: Brain-derived neurotrophic factor
LPS: Lipopolysaccharide
ERK: Extracellular signal-regulated kinase
TLR: Toll-like receptor
Nrf2: Nuclear factor erythroid 2-related factor 2
RIPK: Receptor-interacting protein kinase
MLKL: Mixed lineage kinase domain-like pseudokinase
TXNIP: Thioredoxin-interacting protein
NLRP3: NOD-like receptor thermal protein domain-associated protein 3
IL: Interleukin
TNF-α: Tumor necrosis factor-alpha
NF-κB: Nuclear factor-kappa B
DAMPs: Damage-associated molecular patterns
GABA: Gamma-aminobutyric
AMPA: Alpha-amino-3-hydroxy-5-methyl-4-isoxazole propionate
MAC: Minimum alveolar concentration
rCBF: regional cerebral blood flow
GABAA: γ-Aminobutyric acid sub-type A
MCAO: Middle cerebral artery occlusion
Bax: BCL-2-associated X
ADNP: Activity-dependent neuroprotective protein
ATP: Adenosine triphosphate
SAH: Subarachnoid hemorrhage
VPA: Valproic acid
IFN-γ: Interferon-gamma
Xe-ELIP: Xenon-containing echogenic liposomes
PKC-𝜀: Protein kinase C epsilon
PTSD: Post-traumatic stress disorder
HMGB1: High mobility group box-1
TLR: Toll-like receptor
ARDS: Acute respiratory distress syndrome
IGF-1R: Insulin-like growth factor 1 receptor
HK: Human kidney
PTEN: Phosphatase and tensin homologue
ROS: Reactive oxygen species
EEG: Electroencephalogram
VMAT2: Vesicular monoamine transporter-2
CHI: Cold-head injury
JNK: c-Jun N-terminal kinase
mPTP: mitochondrial permeability transition pore
LIF: Leukemia inhibitory factor
